# The Performance of a Modified Anode Using a Combination of Kaolin and Graphite Nanoparticles in Microbial Fuel Cells

**DOI:** 10.3390/microorganisms12030604

**Published:** 2024-03-18

**Authors:** Lea Ouaknin Hirsch, Bharath Gandu, Abhishiktha Chiliveru, Irina Amar Dubrovin, Shmuel Rozenfeld, Alex Schechter, Rivka Cahan

**Affiliations:** 1 Department of Chemical Engineering, Ariel University, Ariel 40700, Israel; lea.ouaknin@gmail.com (L.O.H.); bharathgandu@gmail.com (B.G.); abhishikthachilivery@gmail.com (A.C.); irinadubrovin@gmail.com (I.A.D.); shmulik2009@gmail.com (S.R.); 2 Department of Environmental Studies, University of Delhi, New Delhi 110007, India; 3 Department of Chemical Sciences, Ariel University, Ariel 40700, Israel; salex@ariel.ac.il; 4 Research and Development Centre for Renewable Energy, New Technologies, Research Centre (NTC), University of West Bohemia, 30100 Pilsen, Czech Republic

**Keywords:** microbial fuel cell, immobilized anodes, kaolin, graphite nanoparticles, *Geobacter sulfurreducens*

## Abstract

The bacterial anode in microbial fuel cells was modified by increasing the biofilm’s adhesion to the anode material using kaolin and graphite nanoparticles. The MFCs were inoculated with *G. sulfurreducens*, kaolin (12.5 g·L^−1^), and three different concentrations of graphite (0.25, 1.25, and 2.5 g·L^−1^). The modified anode with the graphite nanoparticles (1.25 g·L^−1^) showed the highest electroactivity and biofilm viability. A potential of 0.59, 0.45, and 0.23 V and a power density of 0.54 W·m^−2^, 0.3 W·m^−2^, and 0.2 W·m^−2^ were obtained by the MFCs based on kaolin–graphite nanoparticles, kaolin, and bare anodes, respectively. The kaolin–graphite anode exhibited the highest Coulombic efficiency (21%) compared with the kaolin (17%) and the bare (14%) anodes. Scanning electron microscopy and confocal laser scanning microscopy revealed a large amount of biofilm on the kaolin–graphite anode. We assume that the graphite nanoparticles increased the charge transfer between the bacteria that are in the biofilm and are far from the anode material. The addition of kaolin and graphite nanoparticles increased the attachment of several bacteria. Thus, for MFCs that are fed with wastewater, the modified anode should be prepared with a pure culture of *G. sulfurreducens* before adding wastewater that includes non-exoelectrogenic bacteria.

## 1. Introduction

An intensive search is underway for renewable energy sources due to the decrease in fossil fuel resources and the increase in pollution during fossil fuel consumption. One of the most attractive sources of green energy is microbial fuel cell (MFC) technology, which produces electricity through the catabolic activities of electroactive (exoelectrogenic) bacteria. The exoelectrogenic bacteria generate electrons and protons in the anode compartment while oxidizing organic substrates. In the presence of oxygen in the cathode compartment, electrons are reduced to H_2_O. The oxidation and reduction reactions in the MFC drive current production. Although MFCs are promising as an attractive alternative energy source, their use is limited because of their low power production efficiency [1].

Among all the factors, the bacterial anode is the most challenging in the application of MFCs. The electroactive bacterial biofilm on the anode material surface should be stable for a prolonged period and able to transfer its electrons from the distant bacterial layers to the anode material. This study used kaolin and graphite nanoparticles to improve the bacterial anode activity.

Kaolin is an anhydrous aluminosilicate mineral rich in kaolinite (Al_2_Si_2_O_5_(OH)_4_), which consists of repeating layers of Al^3+^ octahedral sheets and Si^4+^ tetrahedral sheets (1:1) stacked one above the other [2,3,4]. It is a low-cost, abundant, and inert mineral [5] with chemical and thermal stability [6] and a high specific surface area [7]. It was also found to be biocompatible [8]. One of the minerals in the kaolinite group is halloysite, with a typical morphology of nanotubes or plates [9]. Lvov et al. examined the biocompatibility of halloysite during in vitro studies of tissues and cells, and they found that halloysite improved cell proliferation [10]. Alekseeva et al. showed that halloysite and a halloysite–magnetite composite increased the viability of *E. coli* by 2–3 fold compared with the control sample (without halloysite or the halloysite–magnetite composite) [8]. Ugochukwu et al. reported that the clay minerals kaolinite and montmorillonite favor bacterial growth and even protect bacteria from heavy metals [11]. Liu et al. used cultured *E. coli* for 15 days in increasing concentrations of polymyxin B, with and without halloysite or kaolinite, and examined the tolerance of the bacteria to antibiotics. It was shown that bacterial cells that were grown in the presence of halloysite or kaolinite were resistant to the antibiotic without significantly damaging the bacterial cell membrane. The results even showed that kaolinite could promote bacterial growth [12].

Carbon nanoparticles are highly conductive and have a large surface area. They can be used as a redox mediator to regulate the electron transfer between electroactive bacteria and the anode material [13,14]. Among carbon nanoparticles, activated carbon and graphite are widely used. Graphite nanoparticles have better conductivity but a lower surface area than activated carbon [15]. Frattini et al. fabricated a graphite–cement composite with graphite up to 80% *w*/*w*. This novel material, which showed high conductivity (2 S·m^−1^) and porosity (60%), was used as an MFC cathode, leading to a maximum power density of around 28 mW·cm^−2^ [16]. Arvaniti et al. developed a graphite–cement composite as the electrode material in an up-flow constructed MFC for wetland water treatment. This MFC was monitored for 7 months. The results showed that, at an organic loading rate of 20.6 g chemical oxygen demand (COD)·m^−2^·d^−1^, the mean power density and Coulombic efficiency were 11.6 ± 3.0 mW·m^−3^ and 0.48 ± 0.22%, respectively [17].

In our previous study, a bacterial anode that was modified by immobilization with kaolin and activated carbon particles produced a maximum power density of 1112 mW·m^−2^ at a current density of 3.33 A·m^−2^, 12% and 56% higher than an anode modified with only kaolin and a bare anode, respectively. *Geobacter* displayed the highest relative distribution (64%) in the biofilm of the anode that was modified with kaolin and activated carbon [18].

In this study, the anode was modified by increasing the biofilm’s adhesion to the anode material surface using kaolin in a combination of graphite nanoparticles to improve the electron transfer from the microorganisms located far away from the anode material. The ratio of the kaolin–graphite concentration was optimized, and the viability of the bacterial anode was examined based on the bacterial enzymes. The bioelectricity of MFCs based on a carbon-cloth anode modified with kaolin, graphite, and *G. sulfurreducens* (a kaolin–graphite anode) was compared with a bacterial anode modified with only kaolin (kaolin anode) and with a bare anode (control). To our knowledge, this is the first study showing a modified anode with a combination of kaolin and graphite nanoparticles.

## 2. Materials and Methods

### 2.1. Inoculation of G. sulfurreducens

*G. sulfurreducens* (DSMZ 12127) was inoculated in *Geobacter* medium (N′ 826, DSMZ Braunschweig, Germany) containing the following components: 1.50 g NH_4_Cl, 0.60 g Na_2_HPO_4_, 0.10 g KCl, 0.82 g Na-acetate, 10.00 mL modified Wolin’s mineral solution (medium 141, 1.50 g Nitrilotriacetic acid, 3 g MgSO_4_ × 7 H_2_O, 0.50 g MnSO_4_ × H_2_O, 1 g NaCl, 0.10 g FeSO_4_ × 7 H_2_O, 0.18 g CoSO_4_ × 7 H_2_O, 0.10 g CaCl_2_ × 2 H_2_O, 0.18 g ZnSO_4_ × 7 H_2_O, 0.01 g CuSO_4_ × 5 H_2_O, 0.02 g AlK(SO_4_)_2_ × 12 H_2_O, 0.01 g H_3_BO_3_, 0.01 g Na_2_MoO_4_ × 2 H_2_O, 0.03 g NiCl_2_ × 6 H_2_O, 0.30 mg Na_2_SeO_3_ × 5 H_2_O, 0.40 mg Na_2_WO_4_ × 2 H_2_O dissolved in 1000 mL of distilled water, DSMZ, Braunschweig, Germany), 10 mL Wolin’s vitamin solution (medium 121, 20 mg Biotin, 20 mg Folic acid, 100 mg Pyridoxine hydrochloride, 50 mg Thiamine HCl, 50 mg Riboflavin, 50 mg Nicotinic acid, 50 mg Calcium D-(+)-pantothenate, 1 mg Vitamin B12, 50 mg p-Aminobenzoic acid, 50 mg (DL)-alpha-Lipoic acid dissolved in 1000 mL of distilled water, DSMZ, Braunschweig, Germany), 8 g Na_2_-fumarate, and 2.50 g NaHCO_3_. The bacterial suspension in a 50 mL borosilicate glass serum bottle with a 20 mm butyl septum was sparged with 80% N_2_: 20% CO_2_. The anaerobic suspension was grown for about 5–7 days until red bacterial aggregates settled on the bottom of the bottle. The bacteria were allowed to settle for an hour without agitation. The liquid portion (supernatant) was poured off, and the concentrated bacterial sediment was collected from several bottles and placed in one bottle. The highly concentrated suspension was agitated for 10 min until most of the bacterial aggregates had been suspended. The optical density (OD) was measured using a GENESYS 10S UV–Visible spectrophotometer (Thermo Scientific, Waltham, MA, USA) at 590 nm. Each of the MFCs was inoculated with 10 mL of *G. sulfurreducens* (1 OD).

### 2.2. Biofilm Viability on Carbon-Cloth Materials

Pure cultures (0.15 OD at 660 nm) of *Bacillus cereus* (DSMZ 31), *Enterobacter cloacae* (DSMZ 30054), and *E*. *coli* (DSMZ 6899) were inoculated in 50 mL tubes with brain heart infusion (BHI) broth (Himedia, India) (37 g dissolved in 1000.00 mL of distilled water) (15 mL) and a 0.5 × 0.5 cm^2^ carbon cloth (ELAT Hydrophilic, FuelCellsEtc, TX, USA), a carbon cloth with kaolin (Sigma-Aldrich, St. Louis, MO, USA), and a carbon cloth with kaolin and graphite nanoparticles (Merck, Darmstadt, Germany). The samples were incubated for 48 h at 37 °C, and then the biofilm on the carbon cloth was gently washed with Dulbecco’s phosphate-buffered saline (Biological Industries, Kibbutz Beit-Haemek, Israel). The biofilm viability was examined using a colorimetric method based on the reduction of tetrazolium salt reagent by bacterial biofilm oxygenases. In this method, a reagent solution of 3-(4,5-Dimethylthiazol-2-yl)-2,5-diphenyltetrazolium bromide (MTT; Merck, Darmstadt, Germany) was added to the tubes, which were then covered with aluminum foil to avoid light penetration. The samples were placed in an incubator for two hours at 30 °C, and the MTT reagent was replaced by a DMSO–Ethanol (1:1) solution, followed by incubation for another 30 min. The absorbance intensity was measured at 540 nm. The same procedure was performed for *G. sulfurreducens*, except for incubation for 7 days at 30 °C in *Geobacter* medium in an anaerobic environment.

### 2.3. MFC Construction and Operation

The experiments were carried out using glass dual-chamber MFCs containing anode and cathode chambers separated by a proton-selective membrane (Nafion^®^ 115; Ionpower, Tyrone, PA, USA). The working volume of the chambers was 200 mL. The anode material (2 × 5 cm) was made of carbon cloth (ELAT Hydrophilic, FuelCellsEtc, Bryan, TX, USA) and was pretreated with cold low-pressure nitrogen plasma to increase its surface hydrophilicity [19]. The anode chamber was inoculated with the anaerobic exoelectrogenic bacterium *G. sulfurreducens* (DSMZ 12127). The kaoline–graphite bacterial anode was constructed in the anode chamber by mixing *G. sulfurreducens* (1 mL of 1 OD at 600 nm), kaolin (12.5 g·L^−1^) [20], and graphite nanoparticles (1.25 g·L^−1^), which after 10 days yielded a paste attached to the 2 × 5 cm carbon cloth. The kaolin anode was made by the same procedure but without adding graphite nanoparticles. In the MFC that utilized the control anode, the carbon cloth was inserted into the anode chamber with only the *G. sulfurreducens* bacteria. In all of the MFCs, the *Geobacter* medium was replaced with a sterile medium after 10 days. Thus, the suspended kaolin and the graphite nanoparticles were removed from the anode chamber. The cathodic chamber contained 200 mM phosphate buffer (13.6 g KH_2_PO_4_ and 23.8 g Na_2_HPO_4_ dissolved in 1000 mL of distilled water). The reference Ag/AgCl electrode (3.0 M KCl, +199 mV vs. SHE; ALS Co. Ltd., Osaka, Japan) was used for electrochemical tests and was placed in the anode chamber. The cathodic electrode (2 × 5 cm) was commercially coated with 0.5 mg·cm^−2^ PtC 60% (Cloth GDE, FuelCellsEtc, Bryan, TX, USA).

The MFC was operated under a constant external resistance of 1000 Ω. The anode chamber was sparged by 80% N_2_:20% CO_2_ gas to ensure anaerobic conditions and was sealed with a screw cap and GL-45 silicone–rubber septa stoppers (SCHOTT AG, Mainz, Germany).

Electrodes were connected via titanium wires. The anode’s contact area and the titanium wires were sealed with epoxy material to ensure electric isolation. An aquarium pump was used to aerate the cathode chamber through a filter (0.45 mm pore size; Whatman, Piscataway, NJ, USA) to provide a sterilized environment. The MFCs were placed in a thermostatic bath at 30 °C. The anode chamber was placed on a magnetic stirrer (120 rpm) for continuous electrolyte stirring. The MFCs were fed with *Geobacter* medium supplied with sodium acetate (10 mM) as the carbon source, which was refreshed when the voltage dropped. The MFCs based on the kaolin–graphite anode, kaolin anode, and control anode were constructed in triplicates.

### 2.4. Chemical Oxygen Demand Assay

Examination of the chemical oxygen demand (COD) was performed by using a kit (Lovibond™ COD tube tests, HR, Great Britain) containing potassium dichromate, sulphuric acid, and metal salts. The samples were mixed gently and incubated in a COD reactor (DBR-001, MRC, Holon, Israel) for two hours at 150 °C. The digested solution was analyzed using a spectrophotometer in the 430–610 nm range (Photometer-system MD 100, Lovibond™, Berlin, Germany).

Coulombic efficiencies were calculated for two cycles at an external resistance of 1000 Ω, based on changes in COD [21], according to Equation (1):

(1) $$C_{E}=\frac{M_{s}\int_{0}^{t_{s}}I\mathrm{d}t}{Fb_{es}v\Delta C}$$

where *M*_s_ is the molecular weight of the substrate (M), Δ*C* is the change in the substrate concentration during the batch cycle time t_b_ (g·L^−1^), *F* is Faraday’s constant, *v* is the volume of the MFC chamber (L), and *b_es_* are the moles of electrons for the substrate (mole).

### 2.5. Electrochemical Analysis

Cyclic voltammetry (CV) and electrochemical impedance spectroscopy (EIS) were performed using a potentiostat (MultiEmStat3+, Palmsens, CL Houten, Vleugelboot, The Netherlands). The CV and EIS tests were carried out by setting the microbial anode as the working electrode, the cathode as the counter electrode, and the Ag/AgCl electrode as the reference electrode. CV was measured at a scan rate of 50 mV·s^−1^ under potentials from −0.8 V to 0.7 V. EIS was done over a frequency range of 200 kHz to 0.01 Hz, and the EIS spectra were fitted by Zview software (ZSimpWin 3.21). The cell voltage was recorded every 10 min during the experiment under an external resistance of 1000 Ω, and the current was calculated using Ohm’s law.

The polarization curve measurement of the different MFCs was performed using the voltage results obtained by applying a range of external resistance (0.1 MΩ–20 Ω) using a decade resistance box and waiting for 10 min at each resistance value to obtain a steady-state voltage.

The current density was calculated using Ohm’s law according to Equation (2):

(2) $$I=\frac{U}{R\times S}$$

where *I* represents the current density in A·m^−2^, *U* is the measured voltage in V, *R* is the external resistance in Ω, and *S* is the projected surface area of the anode electrode in m^2^.

The power density was determined using Equation (3):

(3) $$P=\frac{1000\times U2}{R\times S}$$

where *P* is the power density in W·m^−2^.

### 2.6. Microscopy Analyses of the Bacterial Biofilm Anodes

At the end of the MFC operation, the three different anodes were cut into 0.5 × 0.5 cm^2^ samples and washed with phosphate buffer solution three times. The samples were analyzed using scanning electron microscopy (SEM) and confocal laser scanning microscopy (CLSM). For SEM analysis, the bacterial biofilm anodes were fixed by incubation in Karnovsky’s fixative solution, followed by incubation for 1 h in tannic acid (1%) and incubation in OsO_4_ (4%) for another hour. The samples were washed three times with the phosphate buffer solution (pH 7.2) between each process and then dehydrated in a series of ethanol–water concentrations (volume proportions 30, 40, 50, 60, 70, 80, 90, and 100%) for 10 min each. The samples were air-dried and sputtered with gold before SEM observation in ultra-high resolution (MAIA3 SEM; TESCAN, Brno, Czech Republic) [22,23]. To evaluate the biofilm thickness, the bacterial biofilm anode electrodes were stained with 4′,6-diamidino-2-phenylindole (DAPI) at a concentration of 1 μg·mL^−1^ and washed with PBS to remove the excess stain. The samples were observed under the CLSM with the 10x objective (Zeiss LSM 700 confocal microscope; Carl Zeiss, Weimar, Germany) [24].

## 3. Results and Discussion

The bacterial anode plays a significant role in the MFC’s performance. The anode material should be conductive and biocompatible, while the biofilm on the anode material should be stable and electroactive for a prolonged period. The bacterial anode is one of the main factors limiting the implementation of MFCs. Thus, an effort has been made to improve the bacterial anode’s bio-electroactivity for a prolonged period. Amar Dubrovin et al. encapsulated the bacterial anode in a three-dimensional capsule (2.5 cm in length, 0.8 cm in diameter) to separate the exoelectrogenic biofilm on the carbon cloth anode material from non-exoelectrogenic bacteria in wastewater while enabling the diffusion of nutrients through the capsule membrane [25]. Rozenfeld et al. encapsulated an anode made of a combination of carbon cloth and stainless steel in a dialysis bag with molecular weight cutoffs of 50 kDa, 14 kDa, and 2 kDa [26].

Our study focused on improving the bio-electroactivity of the bacterial anode using kaolin and graphite nanoparticles. The purpose of using kaolin was to promote the Geobacter biofilm’s adhesion to the carbon-cloth anode’s surface. The graphite nanoparticles were added to improve the electron transfer from the bacteria in the biofilm layers lying at a great distance from the anode material. Graphite nanoparticles are known to have better conductivity than activated carbon and a smaller surface area.

### 3.1. The Optimal Graphite Nanoparticle–Kaolin Concentration for Biofilm Formation and Bacterial Anode Electroactivity

**Figure 1:**
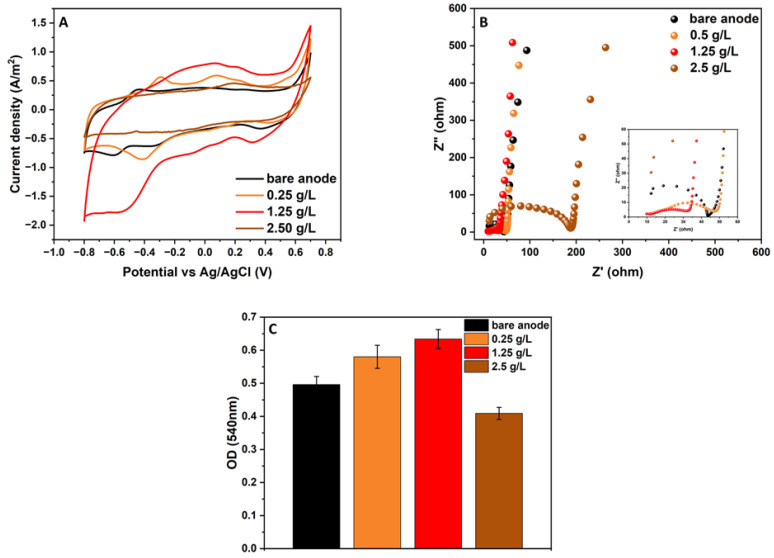
Cyclic voltammetry analysis (**A**), Nyquist plots (**B**), and bacterial anode viability (**C**) of MFCs based on the bare anode (black) and anodes with different graphite concentrations (0.25 g·L^−1^ (orange), 0.125 g·L^−1^ (red), 2.5 g·L^−1^ (brown)).

The optimal combination of graphite nanoparticles and kaolin for biofilm formation and electroactivity was examined by testing MFCs inoculated with different graphite concentrations. At the beginning of the MFC operation, the systems were inoculated with a pure culture of *G. sulfurreducens*, kaolin at a concentration of 12.5 g·L^−1^, and three different concentrations of graphite (0.25, 1.25, and 2.5 g·L^−1^). This experiment lasted for 14 days, and the electroactivity was examined by CV and EIS analyses (Figure 1A,B). The results show that the CV of the modified anode with the graphite nanoparticles at a concentration of 1.25 g·L^−1^ had the largest area curve compared with the other anodes with the graphite concentrations of 0.25 and 2.5 g·L^−1^ and the bare anodes (no graphite or kaolin) (see Figure 1A).

Figure 1B shows the Nyquist plot, which is used to characterize the electron transfer between the electrode and the electrode solution in MFCs. All Nyquist plots appeared as unequally depressed semicircles at high frequencies and showed semi-capacitive behavior at low frequencies. The high-frequency semicircle intercept with the real-part axis of the Nyquist plot represents the solution electrolyte resistance (Rs) of the MFC. At the same time, the depressed semicircle is a series combination of charge transfer resistances and corresponding capacitors in parallel (Rct||C), produced by the biofilm and/or reactive mediators. The Rs of the MFC based on the anode with kaolin and graphite nanoparticles (1.25 g·L^−1^) was 9.82 Ω, the lowest in comparison with the MFCs utilizing kaolin with other levels of graphite nanoparticles (0.25 g·L^−1^, 11.27 Ω; 2.5 g·L^−1^, 12.26 Ω; bare anode, 12.25 Ω). This low Rs suggests that the ratio between kaolin and graphite is essential in providing good connectivity between the graphite conductive particles and the surrounding organic–inorganic biofilm–kaolin matrix that utilizes the additional electrochemical surface area provided by the graphite. Furthermore, the MFC based on the anode with kaolin and graphite nanoparticles (1.25 g·L^−1^) had the best high-frequency Nyquist plot (the smallest semicircle diameter corresponding to a lower Rct) compared with the other anodes with different graphite concentrations (0.25 and 2.5 g·L^−1^) and the bare anode. The EIS results corroborate the analysis of the CV scanning results, showing that the combination of kaolin and graphite nanoparticles (1.25 g·L^−1^) has the highest current, resulting from the electron transfer between the electrogenic bacteria and the graphite in the biofilm on the carbon-cloth anode. At the end of the experiment, the viability of the bacterial anode in the MFCs with the different concentrations of graphite was examined using MTT analysis (Figure 1C). The results indicate that the MFC based on the anode with 1.25 g·L^−1^ graphite led to the highest biofilm viability (0.63 OD at 540 nm), while the bare anode and the anode with 0.25 g·L^−1^ graphite led to biofilm viabilities of 0.49 and 0.58 OD, respectively. It should be noted that the MFC with the highest graphite nanoparticle concentration (2.5 g·L^−1^) showed the lowest biofilm viability (0.41 OD). The results show that the highest graphite concentration (2.5 g·L^−1^) led to the lowest bacterial viability and bio-electroactivity.

### 3.2. The Viability of Different Bacteria on Carbon-Cloth Material in the Presence of Kaolin and Graphite Nanoparticles

**Figure 2:**
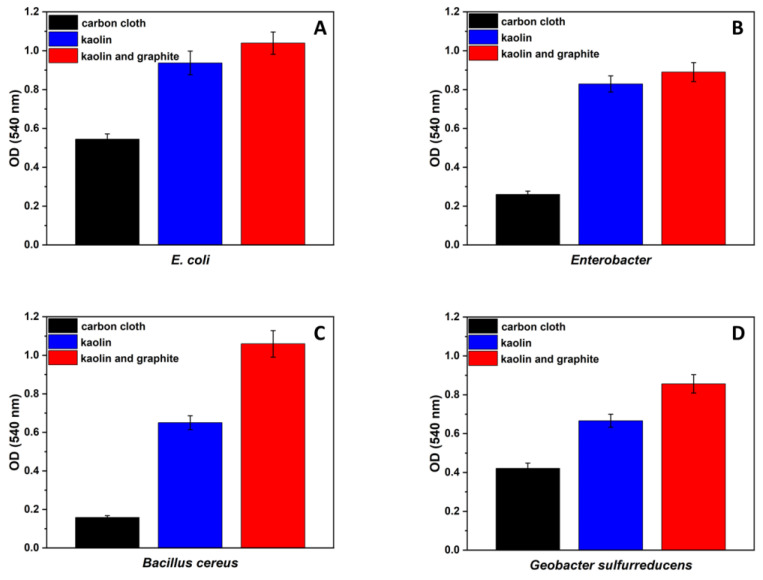
Biofilm viability on carbon-cloth material that was grown in the presence of kaolin and graphite nanoparticles (red), kaolin (blue), and the control (without the addition of kaolin and graphite nanoparticles (black). The different bacteria are *E. coli* (**A**), *Enterobacter cloacae* (**B**), *B. cereus* (**C**), and *G. sulfurreducens* (**D**).

In an attempt to determine whether the increase in bacterial attachment to the carbon-cloth material due to the kaolin is exclusive to *G. sulfurreducens,* other bacteria were suspended with kaolin or with the composite of graphite nanoparticles and kaolin. In this experiment, pure cultures of *Bacillus cereus*, *Enterobacter cloacae,* and *E. coli* were inoculated in tubes containing BHI broth and carbon cloth (0.5 × 0.5 cm^2^). The same procedure was performed but with the addition of kaolin (12.5 g·L^−1^) and kaolin and graphite nanoparticles (1.25 g·L^−1^). The three sets were incubated for 48 h, followed by an analysis of the biofilm viability on each carbon cloth using MTT analysis. To examine the *G. sulfurreducens* biofilm viability, the same experiment was performed, but we changed the medium (*Geobacter* medium)*,* the duration of incubation (7 days), and the environment (anaerobic). The biofilm viability of the different bacteria on the carbon-cloth material is shown in Figure 2.

As demonstrated in Figure 2A–C, the highest biofilm viability in all of the examined bacteria was observed in samples that were grown in the presence of kaolin and graphite nanoparticles. The biofilm viability for *E. coli*, *Enterobacter cloacae*, and *B. cereus* after 48 h of incubation was 1.06, 0.84, and 1.05 OD at 540 nm, respectively. In comparison, the viability of their biofilms on the bare anode was only 0.54, 0.26, and 0.16, respectively. Figure 2D shows that, for the *Geobacter* medium, after 7 days of incubation with kaolin and graphite nanoparticles, the biofilm viability was higher (0.86 OD) compared with the bare (control) anode (0.42 OD).

In summary, it can be seen that the addition of kaolin and graphite nanoparticles improved the biofilm formation compared with the samples where only kaolin was added or the bare control anode. We assume that the addition of graphite nanoparticles supplied a larger surface area for bacterial attachment. The addition of kaolin and graphite nanoparticles may influence all bacterial attachment. Thus, for MFCs supplied with wastewater, the modified kaolin–graphite anode should be prepared with a pure culture of *G. sulfurreducens* before adding wastewater that includes non-exoelectrogenic bacteria.

Kaolin and halloysite embedded with conductive nanoparticles were previously examined for their bacterial biocompatibility. Alekseeva et al. synthesized halloysite with magnetic nanoparticles embedded in the clay mineral matrix. Infrared spectrometry showed the formation of hydrogen bonds between the oxygen-containing groups of magnetite and halloysite. In the presence of halloysite, as well as in the presence of halloysite–magnetite powder, the viability of *E. coli* increased by 2–3 times compared with the control. It was proposed that the viability of *E. coli* was affected by the weak magnetic field of the magnetite particles, which led to the adhesion of the halloysite particles in the immediate surroundings of the bacteria. This adhesion formed a protective framework and improved the physiological processes of the bacteria [8]. Zhu et al. combined kaolin with zero-valent iron nanoparticles to construct a permeable reactive barrier for dechlorinating a trichloroethylene solution. Dechlorinating bacteria of the *Dechloromonas* sp. were added to stabilize the iron nanoparticles and to prevent their loss. This reactive barrier’s trichloroethylene removal rate exceeded 94% for over 365 days [27]. Xu et al. synthesized green kaolin@Fe–Mn binary (hydro)oxides, including iron-oxidizing bacteria composites that possessed a large specific surface area and pore volume as well as an abundance of functional groups, for antimony and arsenic pollution removal. To prepare the composite, a culture of *Acidithiobacillus ferrooxidans* was grown in the presence of kaolin. In this case, the incorporation of bacterial biomass contributed to the binding of metal ions because of bacterial functional groups such as carboxyl, phosphoric, amine, and hydroxyl groups [28]. 

### 3.3. MFC Bio-Electroactivity Performance

#### 3.3.1. Cell Voltage Output Versus Time

Dual-chamber MFC systems were constructed with anodes modified with kaolin–graphite nanoparticles and kaolin only. The electroactivity of these two systems was compared to an MFC utilizing a bare anode. The MFCs were fed with *Geobacter* medium supplemented with acetate as the carbon source and were operated for 40 days under an external resistance of 1 KΩ at 30 °C. During this period, the output voltage was measured using a potentiostat.

**Figure 3:**
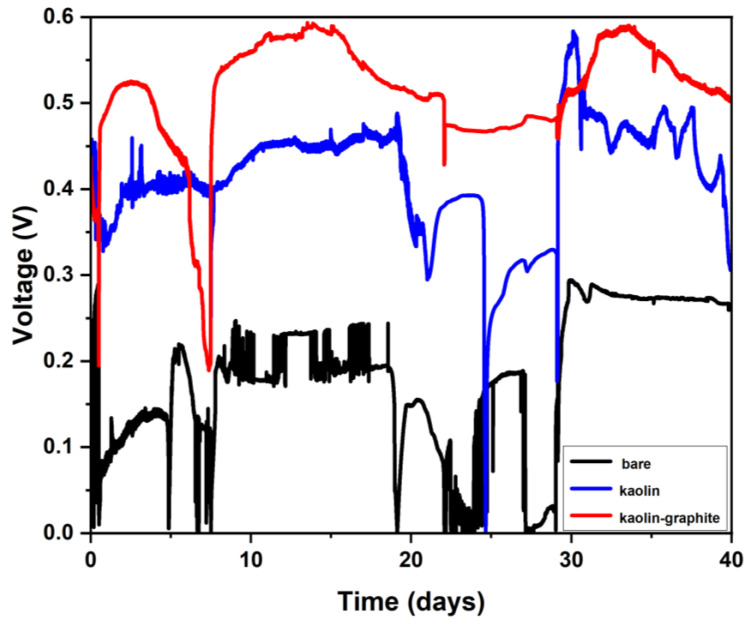
Cell voltage–time curves of the MFCs based on different anodes (kaolin–graphite nanoparticles (red), kaolin (blue), and bare (control) anodes (black)).

As depicted in Figure 3, after three days, the voltage of the MFCs based on kaolin–graphite nanoparticles, kaolin, and bare anodes produced a voltage of 0.52, 0.39, and 0.13 V, respectively. The highest potential, obtained after 30 days of operation, was 0.59, 0.45, and 0.23 V in the MFCs based on kaolin–graphite nanoparticles, kaolin, and bare anodes, respectively. The observed sharp increase in the transition is due to acetate feeding, which abruptly caused an increase in the mediator redox production in the bacterial anode.

We assume that the kaolin improved the attachment of the bacteria, while the graphite nanoparticles increased the anode surface area and facilitated the transfer of electrons from the bacterial cell to the carbon-cloth electrode.

Sayed et al. investigated the electrochemical activity of MFCs based on a composite of graphitic carbon nitride, which was prepared on the surface of a carbon-brush-fiber anode, designated (g-C3N4@CB). Here, the graphitic carbon nitride increased the anode surface area as well as the anode conductivity. They compared it with an MFC based on plain carbon-brush fiber, and the open-circuit voltage (OCV) and anode potentials were recorded until a plateau was observed. The OCVs of the two cells gradually increased over 5 h until they reached steady values of 0.63 and 0.77 V for the carbon-brush-fiber anode and the g-C3N4@CB anode, respectively. An anode potential of 0.27 V vs. Ag/AgCl and an OCV of 0.77 V were obtained by the composite electrode, while an anode potential of 0.1 V vs. Ag/AgCl and an OCV of 0.62 V were obtained by the plain carbon-brush fiber. The MFC based on the composite electrode led to a maximum power density of 772 mW·m^−2^, twelve times that of the MFC based on the carbon-brush-fiber anode [29].

Kim et al. examined the bio-electrochemical activity of MFCs composed of anodes made by the electrodeposition of polydopamine and polypyrrole on a graphite–felt electrode. Polydopamine is characterized by its superior hydrophilicity and adhesive force, which can enhance biofilm formation. Polypyrrole offers electrochemically active sites for bacterial electron transfer. The voltage of the MFC with the composite of polydopamine and polypyrrole on a graphite–felt electrode increased over 33 h. In contrast, the anodes modified with only polydopamine or only polypyrrole and the bare graphite–felt electrodes generated a lower voltage and had a slow startup (~76 h). During the stabilization period, the MFC based on the composite anode with the polydopamine and polypyrrole on the graphite–felt electrode achieved a maximum cell voltage of 600 ± 8 mV, which was 23.5%, 19.6%, and 15.3% higher than the bare graphite–felt electrode, the graphite–felt electrode modified with only polypyrrole, and the graphite–felt electrode modified with only polydopamine, respectively. In addition, the MFC with the composite of polydopamine and polypyrrole on the graphite–felt anode reached 920 mW·m^−2^, which was 1.5, 1.17, and 1.18 times higher than that of the graphite–felt, polydopamine-modified, and polypyrrole-modified anodes, respectively [30].

#### 3.3.2. Power Density and Polarization

**Figure 4:**
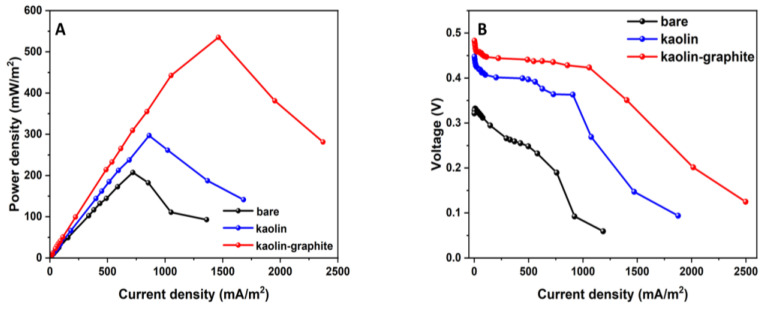
MFC discharge curves for kaolin–graphite nanoparticles (red), kaolin (blue), and bare (control) anodes (black). Power density curves (**A**). Polarization curves (**B**).

Steady-state electrochemical discharge–voltage polarization experiments were carried out after the stabilization of the MFCs under open-circuit conditions for one hour. From Figure 4A,B, it can be seen that the MFC based on the kaolin–graphite nanoparticle anode led to the highest performance, with a maximum power density of 0.54 W·m^−2^ at a current density of 1.46 A·m^−2^, 12% and 56% higher than the kaolin anode (0.3 W·m^−2^ at a current density of 0.86 A·m^−2^) and the bare anode (0.2 W·m^−2^ at a current density of 0.72 A·m^−2^), respectively (Figure 4A). The polarization curves of the MFCs with the modified anodes are shown in Figure 4B. It should be noted that the kaolin–graphite nanoparticle anode displayed a moderate slope, implying the lowest internal resistance of the tested MFCs (0.047 Ω·m^2^), compared with the kaolin anode (0.084 Ω·m^2^) and the bare anode, which showed the sharpest slope (0.18 Ω·m^2^). In summary, the MFC with the kaolin–graphite anode led to the highest current and power density and aligned with the highest biofilm viability (Figure 2D). It can be seen that the MFC based on the modified kaolin–graphite nanoparticle anode led to the highest bio-electroactivity.

Other studies have found improved bio-electroactivity in MFCs with a graphite-modified anode. Mahmoud et al. operated MFCs with three different carbon–felt anodes modified by doping with graphitic mesoporous carbon (2, 5, and 10 mg·cm^−2^ of the anode surface area). These were compared with a control MFC using a pristine carbon–felt electrode. It was observed that the internal resistance of the anodes modified with the graphitic mesoporous carbon was 1.2–2.3 lower than that of the pristine carbon–felt anode, leading to maximum power densities of 70.3, 33.3, and 9.8 mW·m^−2^ for the 10, 5, and 2 mg·cm^−2^-doped anodes, respectively, compared with only 3.8 mW·m^−2^ for the untreated carbon felt [31]. Mukherjee et al. fabricated highly conductive three-dimensional polyvinyl formaldehyde sponges functionalized with acrylamide using polyvinyl alcohol with varying concentrations of graphite nanopowder (0.5, 1, 2.5, 5, and 10 wt%). The performance of these anodes was then evaluated in MFCs. It was shown that the current density attained for all of the studied anodes varied between 10 mA·m^−2^ and 47 mA·m^−2^. The highest current density was recorded for the anode modified with the 10 wt% graphite nanopowder (47.4 mA·m^−2^), while the lowest current density was recorded for the 0.5 wt% graphite nanopowder (10 mA·m^−2^). Similarly, the highest power density (8 W·m^−2^) was recorded for the anode modified with the 10 wt% graphite nanopowder. However, the plain graphite felt led to only 4 W·m^−2^ [32].

#### 3.3.3. Cyclic Voltammetry and Nyquist Plot Measurements during the MFC Operation

**Figure 5:**
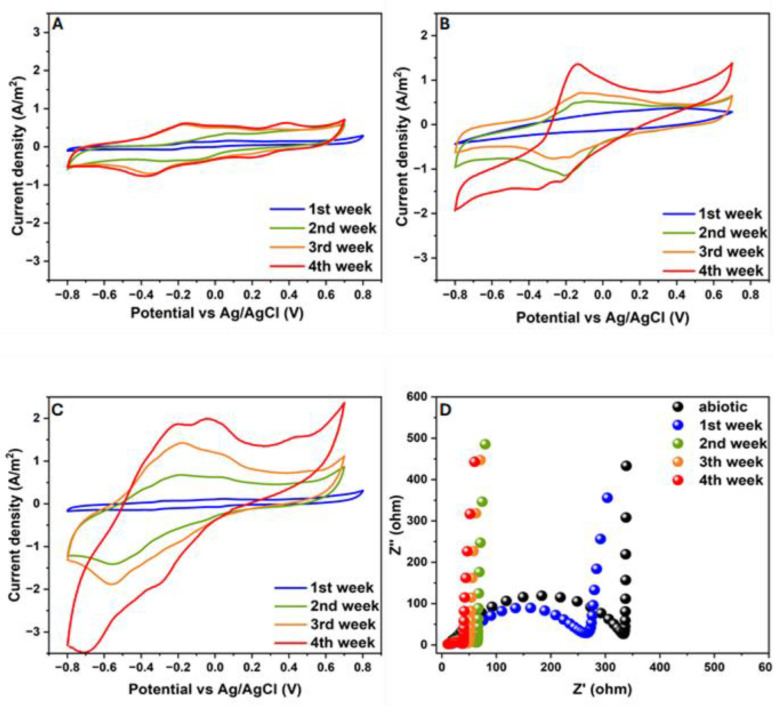
Cyclic voltammograms of the anodes in MFCs based on bare (**A**), kaolin (**B**), and kaolin–graphite (**C**) anodes fed with acetate at a scan rate of 50 mV/s (potentials between −0.8 V and 0.7 V vs. Ag/AgCl). (**D**) Nyquist plots of the MFCs during biofilm formation.

The CV was measured during the MFC operation. The CV voltammograms of the bare anode, the modified kaolin anode, and the modified kaolin–graphite anode are shown in Figure 5A, Figure 5B, and Figure 5C, respectively. The CV of the anode of each MFC was measured after one, two, three, and four weeks. It can be seen that there was a linear correlation between the current density and time for each anode. After one week, a typical redox wave was seen in the curves of all of the MFC bacterial anodes. After four weeks of operation, the peak current density of the kaolin–graphite anode was 1.99 A·m^−2^, which was 1.47 and 3.26 times higher than that of the kaolin anode (1.35 A·m^−2^) and the bare anode (0.61 A·m^−2^), respectively. The increase in current density is ascribed to the enhancement of the catalytic activity on the anode via the improved charge-transfer kinetics resulting from both the graphite nanoparticles and the kaolin embedded in the anode biofilm.

The cyclic voltammograms showed that the kaolin–graphite anode exhibited several redox couples compared with the kaolin anode and the bare anode. We assume that the kaolin and graphite interact with redox intermediate molecules released by the dense bacterial biofilm layer, facilitating a faster charge transfer between the bacteria and the current-collecting carbon electrode.

The electrochemical redox activity increased over time in the oxidation of sodium acetate as can be seen from the voltammograms of the different anodes over time (Figure 5). The CV plots show that, within three weeks of the MFC’s operation (that is, from week 2 to week 4), the main peak of current density increased from 0.15 to 0.6, from 0.25 to 1.38, and from 0.8 to 2 (A·m^−2^) for MFCs with the bare anode, the kaolin anode, and the kaolin–graphite anode.

Figure 5D shows the Nyquist plots of impedance measurements during the MFC operation using the kaolin–graphite modified anode. The anode’s impedance complex profile is depicted before inoculation with the bacteria (measured in an abiotic environment) and throughout the four weeks of *Geobacter* biofilm growth. From the abiotic state to the fourth week of biofilm formation, the Rs decreased from 17.6 to 11.3 Ω. In addition, as seen in Figure 5D, the diameter of the semicircle spectra decreases with time and is likely to be connected to the increased biofilm growth. This part of the plot is ascribed to the kinetic effect of the charge transfer (Rct) from the bacteria to the current-collecting electrode. The results in Figure 5, both in terms of CV and EIS, indicate that the matured biofilm led to higher currents and a lower reaction resistance due to the redox reactions in the anode biofilm.

Other studies have also reported smaller impedance spectra and lower Rct values due to the faster reaction rate or electron transfer dynamic facilitated by the efficient extracellular electron transfer (EET) kinetics at the bacterial cell–electrode interface [33,34,35].

Our results on the measured impedance values demonstrate that the anode resistance during biofilm formation was considerably reduced. This indicates that the EET kinetics between the anode surface and the biofilm had accelerated and that the anode charge-transfer resistance had steadily decreased due to the use of kaolin and graphite nanoparticles. We assume a direct connection exists between the high current density recorded after four weeks of MFC operation and the formation of electroactive biofilm.

Zhu et al. developed a high-performance anode for application in MFCs using heteroatom-doped (N, P, S, Co) porous carbon nanoparticles derived from plant polyphenols. These carbon nanoparticles were reported to be biocompatible and conductive. It was reported that, before biofilm formation, none of the anodes exhibited electrocatalytic activity related to acetate oxidation. However, after one month of operation of the MFC, the electrochemical activity of the modified anode and the control carbon-cloth anode showed typical sigmoidal curves, indicating the electrocatalytic oxidation of sodium acetate by the bacterial biofilm. The peak current density of the modified anode was 10.85 A·m^−2^, far higher than that of the carbon-cloth anode (1.61 A·m^−2^). In addition, the curve area of the modified anode was higher than that of the carbon-cloth anode [36]. Thapa et al. synthetized a carbon xerogel (CX) doped with iron (Fe) and nitrogen (N) followed by modification with graphene oxide (GO) (designated CXFeNGO) and used it as a catalyst for MFCs. When the CXFeNGO-modified electrode was used at the cathode, a maximum power density of 176.5 ± 6 mW·m^−2^ was obtained, 26.8% higher than that of the plain graphite electrode. The power density increased to 48.6% when the pH of the catholyte was increased to 12, producing a power density of 207 ± 4 mW·m^−2^ [37].

### 3.4. COD and Coulombic Efficiency

**Figure 6:**
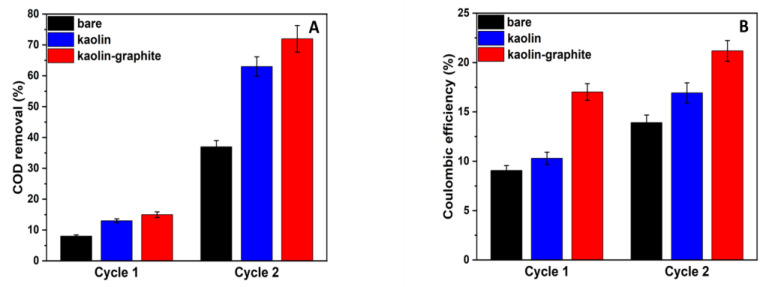
COD removal percentage (**A**) and Coulombic efficiency (**B**) of MFCs based on different anodes (kaolin–graphite nanoparticles (red), kaolin (blue), and bare (control) anodes (black)). Cycles 1 and 2 refer to the 7th and 21st day of MFC operation, respectively.

The COD was examined in the first month of operation of the MFC. The removal of COD during the first and second cycles began on the seventh and twenty-first day of the MFC operation, respectively. Each cycle lasted seven days. As shown in Figure 6A, in all MFCs, there was an upward trend in the during the time of operation. In the second cycle, the MFCs based on the kaolin–graphite anode and the kaolin anode led to COD removal percentages of 72% and 63%, respectively. In comparison, the MFC based on the bare anode led to a COD removal percentage of only 37%.

The Coulombic efficiency was calculated according to Equation (1) and is shown in Figure 6B. The highest Coulombic efficiency was observed for the MFC utilizing the kaolin–graphite anode, followed by the kaolin anode, with the bare anode showing the lowest value. In the second cycle, the kaolin–graphite anode still exhibited the highest Coulombic efficiency (21%) compared with the kaolin anode (17%) and the bare anode (14%).

Marassi et al. evaluated the efficiency of an MFC utilizing a carbon-cloth air cathode with a Pt catalyst and operating in semi-continuous downflow mode. The anode compartment was comprised of three types of electrodes (carbon cloth electrodes, graphite cylinders, and eight graphite rods). The MFC was inoculated with a consortium of *Shewanella oneidensis* and *Clostridium butyricum* and fed with a synthetic medium of an increasing concentration of dairy wastewater. The maximum power density was 3.5 W·m^−3^ at 1.0 A·m^−3^, the COD removal percentage was 89%, and the Coulombic efficiency was 4.5 at the end of the operation [38]. Zhu et al. developed a high-performance anode for application in MFCs using heteroatom-doped (N, P, S, Co) porous carbon nanoparticles derived from plant polyphenols. This MFC led to a power density of 1.72 W·m^−2^ and a current density of 4.52 A·m^−2^, 1.82 times and 1.44 times higher than the values from the MFC with the carbon-cloth anode, respectively. The COD removal percentage in the MFC equipped with the heteroatom-doped porous carbon nanoparticle anode was 94.33%, which was higher than that of the carbon-cloth anode (90.34%). The average Coulombic efficiency of the MFC equipped with the heteroatom-doped porous carbon nanoparticle anode (46.17%) was higher than that of the carbon-cloth anode (36.20%) [36]. Zhang et al. investigated an MFC with graphite electrodes as both the anode and cathode, which was operated with a soil-free anaerobic consortium for phenol degradation. This phenol-degrading MFC showed a Coulombic efficiency of 22.7% [39].

In summary, the MFC equipped with the modified kaolin–graphite anode led to a higher COD removal percentage and Coulombic efficiency compared with the control.

### 3.5. Biofilm Characterization

**Figure 7:**
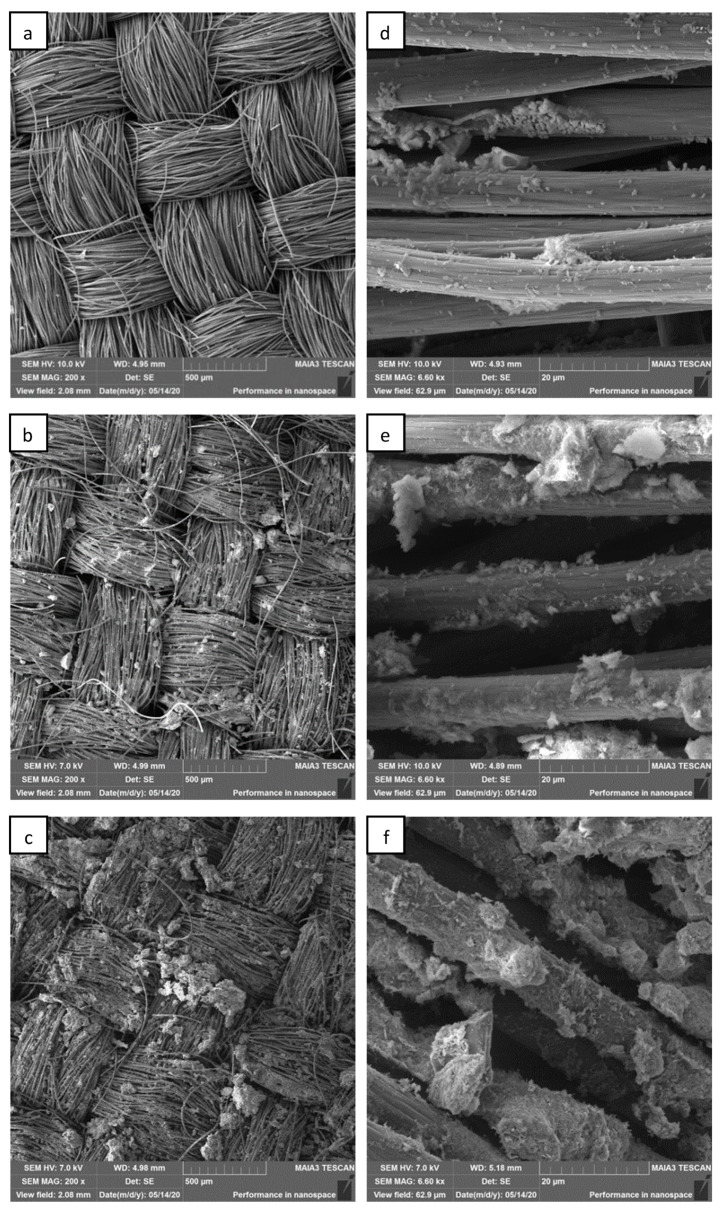
SEM images at a magnification of 200× of (**a**) the bare anode, (**b**) the kaolin anode, and (**c**) the kaolin–graphite anode. (**d**–**f**) represent a further magnified image (6.60 kx) of (**a**), (**b**), and (**c**), respectively.

**Figure 8:**
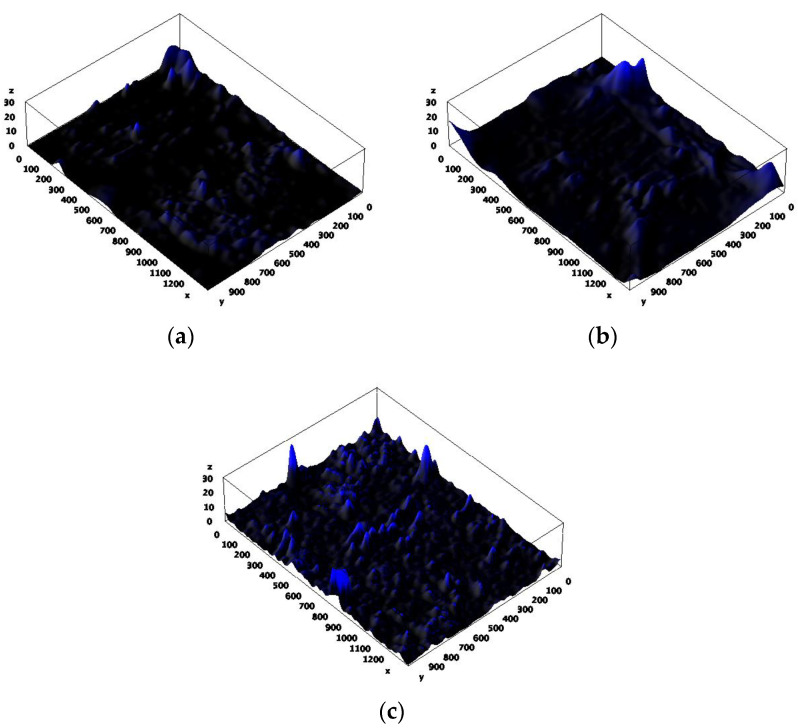
CLSM images at a magnification of 10× of *G. sulfurreducens* biofilm on (**a**) the bare anode, (**b**) the kaolin anode, and (**c**) the kaolin–graphite anode.

After 40 days of operation of the MFC, the biofilm formation on the electrode surface was analyzed by scanning electron microscopy (SEM) and confocal laser scanning microscopy (CLSM). Figure 7a–f show SEM images of the bare anode, kaolin anode, and kaolin–graphite nanoparticle anode, respectively. The SEM images at a magnification of 200× and 6.6 kx clearly show that adding kaolin, composite kaolin, and graphite nanoparticles increased the amount of biofilm compared with the bare anode. Figure 8a–c show CLSM images of the DAPI-stained bacterial anode at a magnification of 10×, which reveal the average height of the biofilm pillars to be 14, 21, and 27 μm for the bare anode, the kaolin anode, and the composite kaolin–graphite nanoparticle anode, respectively. We assume that adding the kaolin and the kaolin and graphite nanoparticles led to a significantly higher degree of biofilm formation compared with the bare anode. These results show that the highest electrochemical redox activity of the MFC equipped with the modified kaolin–graphite anode (Figure 5) is correlated with the biofilm thickness (Figure 7 and Figure 8). This probably affects the charge transfer diffusion.

Godain et al. reported that the initial colonization of the anode by electroactive bacteria may accelerate when the MFC is operated under an applied electrical voltage of 500 mV during the first four days. Metaproteomic/metagenomic analysis showed that the applied voltage during the colonization step predominantly increased the *Geobacter* sp. [40].

Stockl et al. investigated the exopolysaccharide (EPS) secreted by *Geobacter sulfurreducens* cultivated in an MFC based on graphite electrodes under electroactive conditions. The results showed that electroactive cultures secreted significantly more EPSs compared with cells grown under standard heterotrophic conditions (fumarate respiration), and the biofilm was characterized by a wavy and dense structure covering the electrode surface with a thickness ranging between 5 and 10 μm [24]. Schneider et al. isolated exoelectrogenic bacteria from mud samples. SEM images revealed the different adhesion potentials of isolates to the surface of carbon tissue fibers. Eight of the isolates (15%) were able to form massive amounts of biofilm in three days at 23 °C [41]. Engel et al. studied the biofilm characteristics of pure cultures of *Geobacter sulfurreducens* and *Shewanella oneidensis* and compared them to a defined mixed culture of both organisms in bio-electrochemical systems. While pure *S. oneidensis* cultures did not form cohesive and stable biofilms on graphite anodes and only yielded a maximum current density of 0.034 ± 0.011 mA·cm^−2^, pure *G. sulfurreducens* cultures formed biofilms with a thickness of 69 µm and yielded a current of 0.39 ± 0.09 mA·cm^−2^. Compared with the latter, a defined mixed culture of both species was able to yield a 38% higher maximum current density (0.54 ± 0.07 mA·cm^−2^). This increase in current density was associated with a corresponding increase in the thickness of the anodic biofilm to approximately 93 µm [42].

## 4. Conclusions

In this study, a fuel-cell anode was modified by increasing the biofilm’s adhesion to the anode material’s surface and using kaolin in combination with graphite nanoparticles. The optimal graphite nanoparticle and kaolin concentrations for biofilm formation and electroactivity were 12.5 g·L^−1^ and 2.5 g·L^−1^, respectively. The CV of the modified anode with the graphite nanoparticles at a concentration of 1.25 g·L^−1^ had the largest area curve compared with the other anodes with lower graphite nanoparticle concentrations. This modified anode led to the highest biofilm viability (0.63 OD at 540 nm). A potential of 0.59, 0.45, and 0.23 V and a power density of 0.54 W·m^−2^, 0.3 W·m^−2^, and 0.2 W·m^−2^ were obtained by the MFCs based on kaolin–graphite nanoparticles, kaolin, and bare anodes, respectively. The kaolin–graphite anode exhibited the highest Coulombic efficiency (21%) compared with the kaolin anode (17%) and the bare anode (14%). The kaolin–graphite anode exhibited a large amount of biofilm as was shown by SEM and CSLM. We assume that the graphite nanoparticles increased the charge transfer between the distant bacteria and the anode material. It was shown that the addition of kaolin and graphite nanoparticles may influence the attachment of many strains of bacteria. Thus, for MFCs supplied with wastewater, the modified kaolin–graphite anode should be prepared with a pure culture of *G. sulfurreducens* before adding wastewater that includes non-exoelectrogenic bacteria.

## Data Availability

Data are contained within the article.

## References

[1] Logan B.E. (2008). Microbial Fuel Cells.

[2] Unuabonah E.I., Ugwuja C.G., Omorogie M.O., Adewuyi A., Oladoja N.A. (2018). Clays for Efficient Disinfection of Bacteria in Water. Appl. Clay Sci..

[3] Wang Y.H., Siu W.K. (2006). Structure Characteristics and Mechanical Properties of Kaolinite Soils. II. Effects of Structure on Mechanical Properties. Can. Geotech. J..

[4] Yıldız Ozer L., Yusuf A., Uratani J.M., Cabal B., Díaz L.A., Torrecillas R., Moya J.S., Rodríguez J., Palmisano G. (2020). Water Microbial Disinfection via Supported NAg/Kaolin in a Fixed-Bed Reactor Configuration. Appl. Clay Sci..

[5] Oliyaei N., Moosavi-Nasab M., Tamaddon A.M., Fazaeli M. (2019). Preparation and Characterization of Porous Starch Reinforced with Halloysite Nanotube by Solvent Exchange Method. Int. J. Biol. Macromol..

[6] Alipoormazandarani N., Ghazihoseini S., Mohammadi Nafchi A. (2015). Preparation and Characterization of Novel Bionanocomposite Based on Soluble Soybean Polysaccharide and Halloysite Nanoclay. Carbohydr. Polym..

[7] Tharmavaram M., Pandey G., Rawtani D. (2018). Surface Modified Halloysite Nanotubes: A Flexible Interface for Biological, Environmental and Catalytic Applications. Adv. Colloid Interface Sci..

[8] Alekseeva O.V., Smirnova D.N., Noskov A.V., Kuznetsov O.Y., Kirilenko M.A., Agafonov A.V. (2022). Mesoporous Halloysite/Magnetite Composite: Synthesis, Characterization and in Vitro Evaluation of the Effect on the Bacteria Viability. Mater. Today Commun..

[9] Joussein E. (2016). Geology and Mineralogy of Nanosized Tubular Halloysite. Dev. Clay Sci..

[10] Lvov Y., Wang W., Zhang L., Fakhrullin R. (2016). Halloysite Clay Nanotubes for Loading and Sustained Release of Functional Compounds. Adv. Mater..

[11] Ugochukwu U.C., Jones M.D., Head I.M., Manning D.A.C., Fialips C.I. (2014). Biodegradation and Adsorption of Crude Oil Hydrocarbons Supported on “Homoionic” Montmorillonite Clay Minerals. Appl. Clay Sci..

[12] Liu J., Wu P., Wang F., Niu W., Ahmed Z., Chen M., Lu G., Dang Z. (2021). Differential Regulation and the Underlying Mechanisms of Clay Minerals to *Escherichia coli* under the Stress of Polymyxin B: Comparing Halloysite with Kaolinite. Chemosphere.

[13] Wang R., Yan M., Li H., Zhang L., Peng B., Sun J., Liu D., Liu S. (2018). FeS2 Nanoparticles Decorated Graphene as Microbial-Fuel-Cell Anode Achieving High Power Density. Adv. Mater..

[14] Zhang L., He W., Yang J., Sun J., Li H., Han B., Zhao S., Shi Y., Feng Y., Tang Z. (2018). Bread-Derived 3D Macroporous Carbon Foams as High Performance Free-Standing Anode in Microbial Fuel Cells. Biosens. Bioelectron..

[15] Wei J., Liang P., Cao X., Huang X. (2011). Use of Inexpensive Semicoke and Activated Carbon as Biocathode in Microbial Fuel Cells. Bioresour. Technol..

[16] Frattini D., Accardo G., Ferone C., Cioffi R. (2017). Fabrication and Characterization of Graphite-Cement Composites for Microbial Fuel Cells Applications. Mater. Res. Bull..

[17] Arvaniti I., Fountoulakis M.S. (2021). Use of a Graphite-Cement Composite as Electrode Material in up-Flow Constructed Wetland-Microbial Fuel Cell for Greywater Treatment and Bioelectricity Generation. J. Environ. Chem. Eng..

[18] Hirsch L.O., Dubrovin I.A., Gandu B., Emanuel E., Kjellerup B.V., Ugur G.E., Schechter A., Cahan R. (2023). Anode Amendment with Kaolin and Activated Carbon Increases Electricity Generation in a Microbial Fuel Cell. Bioelectrochemistry.

[19] Rozenfeld S., Teller H., Schechter M., Farber R., Krichevski O., Schechter A., Cahan R. (2018). Exfoliated Molybdenum Di-Sulfide (MoS2) Electrode for Hydrogen Production in Microbial Electrolysis Cell. Bioelectrochemistry.

[20] Quintelas C., Rocha Z., Silva B., Fonseca B., Figueiredo H., Tavares T. (2009). Removal of Cd(II), Cr(VI), Fe(III) and Ni(II) from Aqueous Solutions by an *E. Coli* Biofilm Supported on Kaolin. Chem. Eng. J..

[21] Logan B.E., Hamelers B., Rozendal R., Schröder U., Keller J., Freguia S., Aelterman P., Verstraete W., Rabaey K. (2006). Microbial Fuel Cells: Methodology and Technology. Environ. Sci. Technol..

[22] Gandu B., Rozenfeld S., Ouaknin Hirsch L., Schechter A., Cahan R. (2020). Immobilization of Bacterial Cells on Carbon-Cloth Anode Using Alginate for Hydrogen Generation in a Microbial Electrolysis Cell. J. Power Sources.

[23] Fischer E.R., Hansen B.T., Nair V., Hoyt F.H., Dorward D.W. (2012). Scanning Electron Microscopy. Curr. Protoc. Microbiol..

[24] Stöckl M., Teubner N.C., Holtmann D., Mangold K.M., Sand W. (2019). Extracellular Polymeric Substances from Geobacter Sulfurreducens Biofilms in Microbial Fuel Cells. ACS Appl. Mater. Interfaces.

[25] Dubrovin I.A., Hirsch O.L., Rozenfeld S., Gandu B., Menashe O., Schechter A., Cahan R. (2022). Hydrogen Production in Microbial Electrolysis Cells Based on Bacterial Anodes Encapsulated in a Small Bioreactor Platform. Microorganisms.

[26] Rozenfeld S., Gandu B., Hirsch O.L., Dubrovin I., Schechter A., Cahan R. (2021). Hydrogen Production in a Semi-Single-Chamber Microbial Electrolysis Cell Based on Anode Encapsulated in a Dialysis Bag. Int. J. Energy Res..

[27] Zhu J., Zhang L., Liu J., Zhong S., Gao P., Shen J. (2022). Trichloroethylene Remediation Using Zero-Valent Iron with Kaolin Clay, Activated Carbon and Bacteria. Water Res..

[28] Xu R., Li Q., Nan X., Jiang G., Wang L., Xiong J., Yang Y., Xu B., Jiang T. (2022). Simultaneous Removal of Antimony(III/V) and Arsenic(III/V) from Aqueous Solution by Bacteria–Mediated Kaolin@Fe–Mn Binary (Hydr)Oxides Composites. Appl. Clay Sci..

[29] Sayed E.T., Abdelkareem M.A., Alawadhi H., Elsaid K., Wilberforce T., Olabi A.G. (2021). Graphitic Carbon Nitride/Carbon Brush Composite as a Novel Anode for Yeast-Based Microbial Fuel Cells. Energy.

[30] Kim M., Li S., Kong D.S., Song Y.E., Park S.Y., Kim H., Jae J., Chung I., Kim J.R. (2023). Polydopamine/Polypyrrole-Modified Graphite Felt Enhances Biocompatibility for Electroactive Bacteria and Power Density of Microbial Fuel Cell. Chemosphere.

[31] Mahmoud M., El-Khatib K.M. (2020). Three-Dimensional Graphitic Mesoporous Carbon-Doped Carbon Felt Bioanodes Enables High Electric Current Production in Microbial Fuel Cells. Int. J. Hydrogen Energy.

[32] Mukherjee P., Saravanan P. (2020). Graphite Nanopowder Functionalized 3-D Acrylamide Polymeric Anode for Enhanced Performance of Microbial Fuel Cell. Int. J. Hydrogen Energy.

[33] Huang W., Chen J., Hu Y., Chen J., Sun J., Zhang L. (2017). Enhanced Simultaneous Decolorization of Azo Dye and Electricity Generation in Microbial Fuel Cell (MFC) with Redox Mediator Modified Anode. Int. J. Hydrogen Energy.

[34] Ma J., Shi N., Jia J. (2020). Fe_3_O_4_ Nanospheres Decorated Reduced Graphene Oxide as Anode to Promote Extracellular Electron Transfer Efficiency and Power Density in Microbial Fuel Cells. Electrochim. Acta.

[35] Zou L., Huang Y., Wu X., Long Z.-e. (2019). Synergistically Promoting Microbial Biofilm Growth and Interfacial Bioelectrocatalysis by Molybdenum Carbide Nanoparticles Functionalized Graphene Anode for Bioelectricity Production. J. Power Sources.

[36] Zhu K., Wang S., Liu H., Liu S., Zhang J., Yuan J., Fu W., Dang W., Xu Y., Yang X. (2022). Heteroatom-Doped Porous Carbon Nanoparticle-Decorated Carbon Cloth (HPCN/CC) as Efficient Anode Electrode for Microbial Fuel Cells (MFCs). J. Clean. Prod..

[37] Thapa B.S., Seetharaman S., Chetty R., Chandra T.S. (2019). Xerogel Based Catalyst for Improved Cathode Performance in Microbial Fuel Cells. Enzyme Microb. Technol..

[38] Marassi R.J., López M.B.G., Queiroz L.G., Silva D.C.V.R., da Silva F.T., de Paiva T.C.B., Silva G.C. (2022). Efficient Dairy Wastewater Treatment and Power Production Using Graphite Cylinders Electrodes as a Biofilter in Microbial Fuel Cell. Biochem. Eng. J..

[39] Zhang D., Li Z., Zhang C., Zhou X., Xiao Z., Awata T., Katayama A. (2017). Phenol-Degrading Anode Biofilm with High Coulombic Efficiency in Graphite Electrodes Microbial Fuel Cell. J. Biosci. Bioeng..

[40] Godain A., Vogel T.M., Monnier J.M., Paitier A., Haddour N. (2023). Metaproteomic and Metagenomic-Coupled Approach to Investigate Microbial Response to Electrochemical Conditions in Microbial Fuel Cells. Microorganisms.

[41] Schneider G., Pásztor D., Szabó P., Kőrösi L., Kishan N.S., Raju P.A.R.K., Calay R.K. (2023). Isolation and Characterisation of Electrogenic Bacteria from Mud Samples. Microorganisms.

[42] Engel C., Schattenberg F., Dohnt K., Schröder U., Müller S., Krull R. (2019). Long-Term Behavior of Defined Mixed Cultures of *Geobacter Sulfurreducens* and *Shewanella Oneidensis* in Bioelectrochemical Systems. Front. Bioeng. Biotechnol..

